# Aspartame Not Linked to Cancer

**DOI:** 10.1289/ehp.115-a16

**Published:** 2007-01

**Authors:** Eyassu G. Abegaz

**Affiliations:** Aginomoto Corporate Services LLC, Washington, DC, E-mail: abegaze@ajiusa.com

In an article published in the March 2006 issue of *Environmental Health Perspectives* (*EHP*) Soffritti et al. (2006) of the European Ramazzini Foundation of Oncology and Environmental Sciences (ERF) reported that aspartame was associated with an increase in lymphomas and leukemias, transitional cell carcinomas of the renal pelvis and urether, malignant schwanomas of peripheral nerves, and hyperplasia of the olfactory epithelium.

After the publication of the ERF aspartame study (Soffritti et al. 2006), the European Commission asked the European Food Safety Authority (EFSA) to assess the ERF aspartame carcinogenicity study results as a matter of high priority following the publication (EFSA 2005). The EFSA’s Scientific Panel on Food Additives, Flavorings, Processing Aids and Materials in Contact with Food (AFC), an 18-member panel that consisted of independent regulatory scientists and toxicologists, assessed the ERF aspartame carcinogenicity study using not only the ERF publication but also more extensive primary data and reports provided by ERF (EFSA 2006). Concurrently, the U.K. Food Standards Agency requested the opinion of the U.K. Committee on Carcinogenicity of Chemicals in Food, Consumer Products and Environment (COC) on the quality, analysis, and interpretation of the results of the ERF aspartame carcinogenicity study (Soffritti et al. 2006).

After a lengthy evaluation process, on 5 May 2006, the EFSA published a 44-page report (EFSA 2006). A summary comment of the EFSA report on ERF study included the following:

The increased incidence of lymphomas/leukaemias reported in treated rats was unrelated to aspartame, given the high background incidence of chronic inflammatory changes in the lungs and the lack of a positive dose–response relationship. … The slight increase in incidence of these tumours in rats fed aspartame is considered to be an incidental finding of the ERF study and can therefore be dismissed. (EFSA 2006)

The preneoplastic and neoplastic lesions of the renal pelvis, ureter and bladder occurring primarily in female rats along with renal calcification were most probably treatment-related, at least at the higher doses. It is widely accepted that the effect is a high dose effect of irritant chemicals or chemicals producing renal pelvic calcification as a result of imbalances in calcium metabolism, specific to the rat. The Panel considers that these effects are of no relevance for humans. (EFSA 2006)

The data on total malignant tumours do not provide evidence of a carcinogenic potential of aspartame. … [T]he aggregation of all malignant tumour incidences or all malignant tumour-bearing animals for statistical purposes is not justified, given that, as explained above, the lymphomas/ leukaemias and the renal tumours should have been excluded from the analysis. (EFSA 2006)

Concerning the malignant schwannomas, … the numbers of tumours were low, the dose–response relationship, while showing a positive statistical trend in males, was very flat over a wide dose range and there is also uncertainty about the diagnosis of these tumours. … [T]his finding can only be fully evaluated following a histopathological peer-review of all relevant slides related to the nervous system in the ERF study and if necessary also from the historical controls. (EFSA 2006)

Furthermore, the COC’s March 2006 minutes on the publication of the ERF aspartame study (Soffritti et al. 2006) concluded,

… [I]n view of the problems in the design of the study and some concerns about the microbiological status of the colony, it was not possible to draw conclusions about the potential carcinogenicity of aspartame from the results.

The study by Soffritti et al. (2006) has major flaws that bring into question the validity of the findings. Its publication in *EHP* is not without consequence to the reputation of the National Institute of Environmental Health Sciences or to the health of the U.S. public. Publication of invalid and misleading research results relating to products such as aspartame, which can be of benefit in the battle against obesity and have a history of safe use, are a disservice to the tax-paying citizens of the United States.
